# A Case of Crural Hernia Which Terminated in Gangrene, and in Which the Fæces Were Discharged by the Opening in the Groin, but Which Was Afterwards Healed, and the Fæces at Last Voided by the Natural Passage

**Published:** 1808-06

**Authors:** Neh. Duck

**Affiliations:** Crewkerne


### *A Case of Crural Hernia which terminated in Gangrene, and in which the Fæces were discharged by the opening in the Groin, but which was afterwards healed, and the Fæces at last voided by the natural Passage.*

ELIZABETH CROCKER, housekeeper to Joseph Greenham, gent. at Chisselborough, aged 50, having been subject to Crural Hernia on the right side about a year and a half, was seized on the 19th of August, 1803, (after using considerable exertions in her domestic avocations,) with those symptoms which usually denote strangulation of the intestine. I was sent for to her on the 21st, when I found her in excruciating pain, anxiety, and restlessness, accompanied with singultus and incessant vomiting; there was also great inflammation of the integuments with enlargement of the inguinal glands, under which I could feel the protruded portion of the intestine. I attempted the reduction by pressure, after having taken from the arm about sixteen ounces of blood; but not succeeding, and seeing the dangerous tendency of the case, I requested that Dr. Proctor might be sent for. When Dr. P. arrived, he recommended a turpentine clyster to be injected, and immersion in the warm bath, which produced no relief. After coming out of the bath, another clyster of an infusion of two drachms of nicotiana in a pint of water was administered, which brought on a degree of deliquium and general relaxation of the system, and was soon followed by two copious evacuations by stool: she then felt easy, and we viewed the case in a more favourable light, but at the same time there was a frequency and smallness of the pulse, which indicated some danger; saline draughts with tinct. opii were prescribed to be taken at intervals during the night. On the following morning we visited her, and found she had continued free from pain in the bowels, but there was not any amendment in the state of the pulse, and the inguinal glands continued painful and inflamed, to which a solution of muriated ammonia in vinegar and water was directed to be applied and frequently renewed; the saline medicine was co tinued. On the 23d, the symptoms were much the same as yesterday, and as there had been no evacuation by the bowels, an aperient medicine was ordered her, which produced a proper discharge by stool. When I saw her on the 24th, I discovered that the integuments had given way, and that a portion of sphacelated gut had been thrown off, of about five inches in length,

 followed followed by a discharge of fæces. I did not particularly examine the gangrenous part which had come away at that time, but requested it might be preserved, that Dr. Proctor and myself might inspect it together; but before we met, it had, through inattention, been thrown into the vault.

We now considered it a lost case, and soon expected to hear of her dissolution; she was, however, directed to have small quantities of nourishing diet given at frequent intervals, and to be kept perfectly quiet. For many days the fæces were entirely discharged by the opening in the groin, when the wound put on a promising appearance. The fæces were then discharged partly by the anus, and in part through the crural passage, through which there was a frequent gurgling of air. By pursuing the above plan, her strength was supported, and health returned gradually. The ulceration healed in about six months, so far as to prevent the exit of the contents of the bowels, and was shortly after completely cicatrized, and has continued so, the patient having the alvine evacuations by the natural passage, and is now living at Chisselborough in perfect health, in the same place of service.

I am aware that this is not a solitary case of gangrene of the bowels terminating in so favourable a way, but instances of the kind are very rare, and *this* was judged of sufficient importance by Dr. Proctor and myself, to warrant its being submitted to the perusal of the Western Medical Society. A case, similar to the one related above, is recorded in the third volume of Medical Observations and Inquiries, by a Society of Physicians in London, in a note to Article VIII. in these words.

"Abraham Pike, aged about 30, had laboured under a Hernia Intestinalis for several years, which was one day in the year 1731 increased to such a degree by overheaving himself, in carrying water to extinguish a fire, that he could not at night reduce it as usual. Being without medical assistance, he suffered for about a fortnight terrible pains, with constipation from a strangulation of the intestine. At the end of that time, Mr. Cookesley first saw him, and found the parts in a state of mortification. He cut away (as he imagined) six inches of the whole annular substance of the intestine, and nearly half the scrotum, including, as he thought, the spermatic vessels and testicle of that side. The excrements were entirely discharged by the wound for some time; but as this contracted, that discharge lessened, and in about a month they passed solely by the anus."

Sharp,

Sharp, in his Treatise on the Operations of Surgery, observes, "It rarely happens that patients submit to this incision before the gut is mortified, and it is too late to do service: not but that there are instances of people surviving small gangrenes, and even *perfectly recovering afterwards*. I myself have been a witness of the cure of two patients who some time after the operation, when the eschar separated, discharged their fæces through the wound, and continued to do so for a few weeks in small quantities, when at length the intestine adhered to the external wound, and then was *fairly heale*d."

He also observes, "These cases, however, are only mentioned to furnish Surgeons with the knowledge of the possibility of such events, and not to mislead them so far as to make favourable inferences with regard to gangrenes of the bowels, which generally are mortal."

NEH. DUCK.

*Crewkerne, June* 15, 1807.

